# Hypercalcemia and huge splenomegaly presenting in an elderly patient with B-cell non-Hodgkin's lymphoma: a case report

**DOI:** 10.1186/1752-1947-4-330

**Published:** 2010-10-19

**Authors:** Ali AM Ghazi, Hamid Attarian, Shirin Attarian, Abolghasem Abasahl, Ebrahim Daryani, Ebrahim Farasat, Marina Pourafkari, Farrokh Tirgari, Siavash M Ghazi, Kalman Kovacs

**Affiliations:** 1 Research Institute for Endocrine Sciences, Taleghani General Hospital, Shahid Beheshti University of Medical Sciences, Tehran, Iran; 2 Department of Hematology and Oncology, Taleghani General Hospital, Shahid Beheshti University of Medical Sciences, Tehran, Iran; 3 Department of Surgery, Imam Khomeini Hospital, Tehran University of Medical Sciences, Tehran, Iran; 4 Department of Internal Medicine, Imam Khomeini Hospital, Tehran University of Medical Sciences, Tehran, Iran; 5 Department of Cardiology, Sina Hospital, Tehran University of Medical Sciences, Tehran, Iran; 6 Department of Radiology, Taleghani General Hospital, Shahid Beheshti University of Medical Sciences, Tehran, Iran; 7 Department of Pathology, Imam Khomeini Hospital, Tehran University of Medical Sciences, Tehran, Iran; 8 Department of Laboratory Medicine, Saint Michael's Hospital, University of Toronto, Ontario, Canada

## Abstract

**Introduction:**

Hypercalcemia is the major electrolyte abnormality in patients with malignant tumors. It can be due to localized osteolytic hypercalcemia or elaboration of humoral substances such as parathyroid hormone-related protein from tumoral cells. In hematological malignancies, a third mechanism of uncontrolled synthesis and secretion of 1-25(OH)_2_D_3 _from tumoral cells or neighboring macrophages may contribute to the problem. However, hypercalcemia is quite unusual in patients with B-cell non-Hodgkin's lymphoma.

**Case presentation:**

An 85-year-old Caucasian woman presented with low grade fever, anorexia, abdominal discomfort and fullness in her left abdomen for the last six months. She was mildly anemic and complained of fatigability. She had huge splenomegaly and was hypercalcemic. After correction of her hypercalcemia, she had a splenectomy. Microscopic evaluation revealed a malignant lymphoma. Her immunohistochemistry was positive for leukocyte common antigen, CD20 and parathyroid hormone-related peptide.

**Conclusion:**

Immunopositivity for parathyroid hormone-related peptide clearly demonstrates that hypersecretion of a parathyroid hormone-like substance from the tumor had led to hypercalcemia in this case. High serum calcium is seen in only seven to eight percent of patients with B-cell non-Hodgkin's lymphoma, apparently due to different mechanisms. Evaluation of serum parathyroid hormone-related protein and 1-25(OH)_2_D_3 _can be helpful in diagnosis and management. It should be noted that presentation with hypercalcemia has a serious impact on prognosis and survival.

## Introduction

Hypercalcemia is the major electrolyte abnormality in patients with malignant tumors. It can be due to skeletal invasion, known as localized osteolytic hypercalcemia or elaboration of humoral substances such as parathyroid hormone-related protein (PTHrP) from tumoral cells. In hematological malignancies, a third mechanism, uncontrolled synthesis and secretion of 1-25(OH)_2_D_3 _from tumoral cells or neighboring macrophages, may contribute to the problem [1,2].

Hypercalcemia is common in patients with hematological malignancies. About 30% of patients with multiple myeloma and 60% of patients with T-cell non-Hodgkin's lymphoma (NHL) experience hypercalcemia due to osteolytic mechanisms or PTHrP hypersecretion respectively. By contrast, hypercalcemia is seen in only seven to eight percent of patients with B-cell NHL [3], mostly due to uncontrolled endogenous production of 1-25(OH)_2_D_3 _from tumor cells. Hypercalcemia that is secondary to elaboration of PTHrP in patients with B-cell NHL is quite unusual and, according to the best of our knowledge, limited numbers of such patients have been observed [3-7].

In our case report, we present the case of an 85-year-old Iranian woman who had huge splenomegaly and hypercalcemia. She was finally proven to have a PTHrP-producing B-cell lymphoma of her spleen.

### Case presentation

An 85-year-old Iranian, Caucasian woman presented with low grade fever, anorexia, abdominal discomfort and fullness in her left abdomen for the last six months. An abdominal computed tomography (CT) scan performed six months previously revealed a filling defect in her spleen, which was interpreted as a splenic cyst. No specific treatment was done at that time.

**Figure 1:**
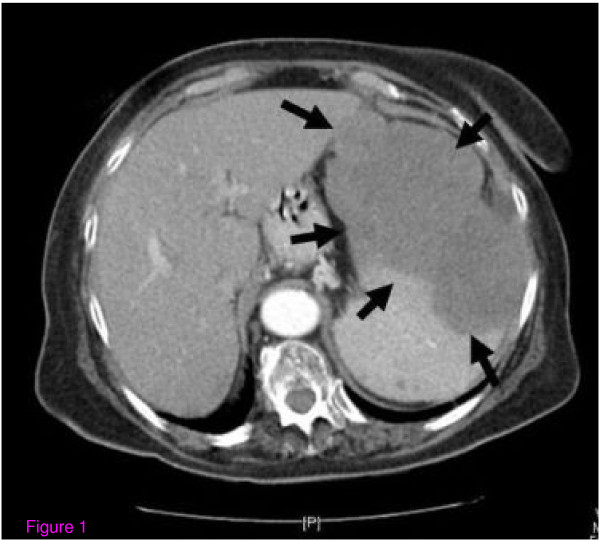
**An abdominal CT scan of the patient during the first hospital admission**.

On examination, she was mildly anemic and complained of fatigability. On abdominal examination a markedly enlarged spleen was palpated. No peripheral lymphadenopathy was noted. Table 1 shows her laboratory data. Unfortunately, her PTHrP measurement was not available to us. Her chest and mediastinal CT scan was unremarkable except for some fibrotic changes compatible with her age. No mediastinal lymphadenopathy was seen. In her abdominal CT scan, it was noted that her spleen was large and that it contained a definite mass occupying about two thirds of the splenic space. No abdominal or para-aortic lymph nodes were seen (Figure 1).

**Table 1:** Laboratory data of the patient on admission

	Patient	Normal range
Hb	11.4	12-14 g/dl
WBC	8.1*10/ml	4-10.8 × 10^3/^ml
Ca	13.3	8.5-10.3 mg/dl
P	3.5	2.5-4.5 mg/dl
Creatinin	1.9	0.5-1.2 mg/dl
PTH	15	15-65 pg/ml
LDH	936	<480 IU/L
25OH D_3_	8.6	<30 ng/ml
1-25(OH)_2_D_3_	12.7	20-70 pg/ml
24 h Urine Calcium	208	<120 mg/24 h
ESR	62	6-20 mm

**Figure 2:**
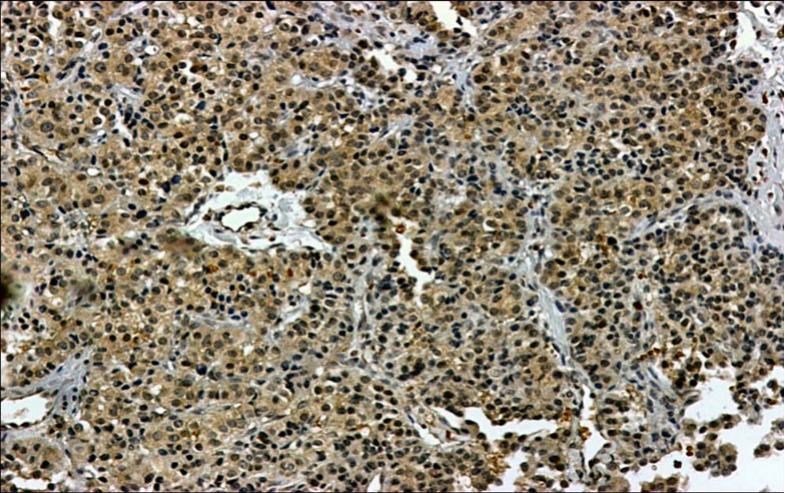
**PTHrP immunostaining**.

**Figure 3:**
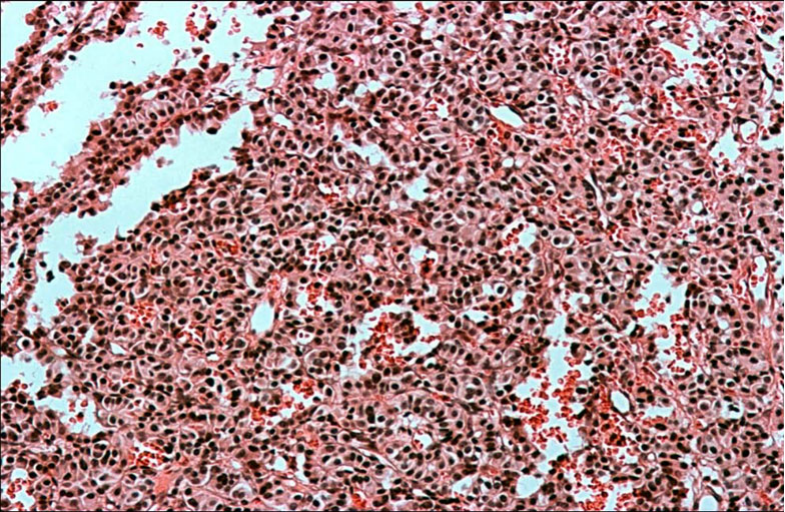
**H&E staining (hematoxylin and eosin staining)**.

**Figure 4:**
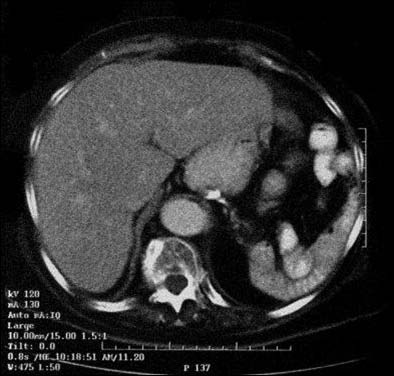
**A CT scan of the patient one year after surgery**.

Her serum calcium was gradually corrected by the use of intravenous saline and furosemide over the next few days. She did not receive any other specific treatment for her hypercalcemia (such as calcitonin or bisphosphonates). On the fifth day of her admission, she underwent a total splenectomy and a huge spleen measuring 22 × 18 × 14 cm, weighing 1800 grams and harboring a firm mass was extracted. Microscopic evaluation revealed a high-grade malignant lymphoma with foci of necrosis (Figure 2). Her immunohistochemistry was positive for LCA, CD20, and PTHrP (Figure 3). After surgery her serum calcium levels were 8.5-9.6 mg/dl but her low grade fever and anorexia resumed. A bone marrow biopsy was performed and there was no bone marrow involvement. Based on the lack of lytic bone lesions, no bone marrow involvement, no plasmacytosis in her bone marrow, and the lack of gammopathy in her serum protein electrophoresis, other hematological malignancies, including multiple myeloma, were ruled out. She was treated with six courses of R-CHOP. Based on her age (85-years-old), weight (70 kg), height (1.58 m) and body surface area (1.7 m^2^), the dosage of the chemotherapy regimen was as follows: 350 mg/m^2 ^of rituximab for a total dose of 600 mg, 600 mg/m^2 ^of cyclophosphamide for a total dose of 1000 mg, 40 mg/m^2 ^of Adriamycin (doxorubicin) for a total dose of 70 mg, 1.4 mg/m^2 ^of Oncovin (vincristine) for a total dose of 2 mg per injection and 75 mg of prednisolone daily for five days. After the second course of chemotherapy, her general condition improved, her fever disappeared and her appetite resumed. Five months after therapeutic courses, there were no clinical or laboratory signs of disease. Figure 4 shows an abdominal CT scan performed one year after surgery.

## Discussion

Immunohistochemistry immunopositivity for PTHrP clearly demonstrates that hypersecretion of the PTH-like substance from the tumor had led to hypercalcemia in this case. Contrary to Adult T-cell leukemia/lymphoma (ATLL) in which hypercalcemia is common and almost always secondary to PTHrP hypersecretion, high serum calcium is seen in only seven to eight percent of patients with B-cell NHL, apparently due to different mechanisms. Majumdar in his study on 112 patients with B-cell NHL showed that eight patients (7.1%), six with high grade and two with low grade disease had elevated serum calcium levels [8]. Most patients had stage 3 and 4 (Stage 3: NHL is in two lymph node groups, with/without partial involvement of an extranodal organ or site above and below the diaphragm. Stage 4: NHL is extensive (diffuse) in one organ or site, with/without NHL in distant lymph nodes.) and survived between two to 11 months after the appearance of hypercalcemia. No explanation about the etiology of hypercalcemia was given in that paper.

The first study that linked elevated serum calcium to hypersecretion of PTHrP from the tumoral cells belongs to Wada *et al *[9]. In their study about a 40-year-old man with B-cell NHL, the authors demonstrated not only high serum levels of PTHrP, but also the parallel changes in serum calcium and PTHrP during a course of therapy. They also demonstrated the presence of immunoreactive PTHrP in the tumor extract and proved the bioactivity of the tumor extract producing C-AMP in osteoblasts. Since that time, a limited number of patients with hypercalcemic B-cell NHL secondary to PTHrP have been reported [6,8-15]. Table 2 shows the clinical and laboratory data of 10 such patients, including ours. As shown in Table 2, the hypercalcemia was severe and life-threatening and immediate therapeutic modalities such as forced hydration and application of furosemide, calcitonin and pamidronate were undertaken to alleviate the problem.

**Table 2:** Clinical and laboratory data of B-cell NHL patients with hypercalcemia due to PTHrP hypersecretion

Number	Age (year)	Gender	Ca mg/dl	PTHrP Pmol/L*	LDH Iu/L	1-25(OH)_2_D_3 _Pg/ml	Outcome	Author, Year
1	40	male	18.2	310 (21.8-44.8)	2349	41	died after 3 months	Wada *et al*, 1992
2	64	female	16	151 (13.8-55.3)	1750	Normal		Hamihara *et al*, 1996
3	70	female	26.3	10.3 (<2.5)	-	<20		Ranganath *et al*, 1998
4	49	female	16.2	52 (<16)	1795	20.5	died after 2 months	Uno *et al*, 1998
5	73	male	17	1.3 (<0.5)	-	Normal	partial improvement	Daroszewski *et al*, 1999
6	52	male	18.6	8 (<0.8)	-	-	partial improvement	Knobel *et al*, 2001
7	93	female	16.6	5 (<0.6)	-	-	died	Ota, 2003
8	69	male	18.8	13 (<1.3)	356	47	died at hospital	Schottker *et al*, 2006
9	50	female	18.3	6.2 (<0.6)	433	17	died at hospital	Takasaki *et al*, 2006
10	85	female	13.3	NA	936	12.8	alive	Ghazi *et al*, 2008

Serum PTH and 1-25(OH)_2_D_3 _were low in most cases due to suppression of the parathyroid glands and renal α-hydroxylase secondary to hypercalcemia.

It is also evident that hypercalcemia is a manifestation of advanced disease and, as with other cases of humoral hypercalcemia of malignancy (HHM), points to a poor prognosis. All the patients, except our patient who is still in remission, died between two to 11 months after the appearance of hypercalcemia.

Uncontrolled synthesis of 1-25(OH)_2_D_3 _as the etiology of hypercalcemia has also been described in patients with B-cell NHL [6,16-18].

## Conclusions

We conclude that although hypercalcemia is rare in patients with B-cell NHL, it should be properly diagnosed and urgently treated. The evaluation of serum PTHrP and 1-25(OH)_2_D_3 _can be helpful in diagnosis and management. It should also be noted that presentation with hypercalcemia has a serious impact on prognosis and survival.

## Consent

Written informed consent was obtained from the patient for publication of this case report and any accompanying images. A copy of the written consent is available for review by the Editor-in-Chief of this journal.

## Competing interests

The authors declare that they have no competing interests.

## Authors' contributions

AG analyzed and interpreted data regarding our patient's endocrine disease and hypercalcemia. HA analyzed and interpreted data regarding her hematologic disease and performed her chemotherapy. SA carried out data collection, was a major contributor in the writing of the manuscript and coordinated all members of the group. AA performed splenectomy on our patient. ED performed the gastrointestinal work up. EF undertook cardiovascular management before the surgery. MP analyzed and interpreted all X-rays and abdominal CT scans. FT perfomed, analyzed and interpreted the pathological specimens resulting from her lymph node, spleen, bone marrow, and all immunohistochemical studies. SG contributed to writing the manuscript and the collection of data. KK undertook some laboratory analysis and endocrine interpretation. All authors read and approved the final manuscript.
